# Response to furmonertinib in a patient with non-small cell lung cancer harboring HER2 exon 21 insertion mutation: a case report

**DOI:** 10.3389/fonc.2024.1440379

**Published:** 2024-10-28

**Authors:** Chunxiao Ni, Ling Zhang, Xin Yu, Yu Pang, Jiaju Xu

**Affiliations:** ^1^ Department of Minimally Invasive Oncology, Tai’an City Central Hospital, Tai’an, Shandong, China; ^2^ Department of Medical Oncology, Tai’an City Central Hospital, Tai’an, Shandong, China; ^3^ Department of Spine Surgery, Tai’an City Central Hospital, Tai’an, Shandong, China; ^4^ Department of Pathology, Tai’an City Central Hospital, Tai’an, Shandong, China

**Keywords:** furmonertinib, non-small cell lung cancer, HER2, mutation, case report

## Abstract

**Background:**

This is the first case report describing a patient with non-small cell lung cancer (NSCLC) harboring two rare human epidermal growth factor receptor 2 (HER2) exon 21 insertion mutations, who responded to furmonertinib treatment. Furmonertinib maybe one effective and economical treatment for NSCLC patients harboring HER2 mutations with minor side effects.

**Case description:**

We present a case report of a 49-year-old female diagnosed with stage IV lung adenocarcinoma who complained of irritating dry cough symptoms followed by chest tightness. Firstly, we describe the patient’s treatment history, including failed third-line combination treatments of systemic chemotherapy with bevacizumab or carrelizumab or anlotinib, primary lung tumor recurrence, bilateral lung metastases progression, and new brain metastatic lesion detection. Next, we detail the patient’s fourth-line treatment with radiotherapy for brain metastases and two cycles of bevacizumab plus Abraxane and cisplatin, however, the disease progressed and relapsed. After that, comprehensive genomic profiling revealed two HER2 exon 21 insertion mutations. Subsequently, the patient received targeted therapy with furmonertinib and achieved 11 months of progression-free survival. The patient received pyrrotinib therapy for 2 months after disease progression, but the disease continued to progress. In October 2023, the patient received therapy with furmonertinib again, and a month later, the disease went into partial remission. However, the patient died due to hypoproteinemia combined with severe pneumonia in December 2023.

**Conclusion:**

Furmonertinib may be effective for NSCLC patients with HER2 T8962A and L869R mutations and further studies are needed to confirm these results in prospective clinical trials.

## Background

HER2 alterations in NSCLC are mainly caused by HER2 protein over-expression, gene amplification, and mutation (1, 2). Their incidence rates in NSCLC are 7.7%-23%, 2%-22%, and 1%-6.7%, respectively, considered generally markers of poor prognosis (2–7). HER2 mutations are predominantly observed in female patients, never smokers, and patients with adenocarcinoma subtypes (5, 8).

Across all cancers, HER2 mutations occurred most frequently in the tyrosine kinase domain (TKD) (46%), which included mutations in exon 20 (20%), exon 19 (11%), and exon 21 (9%). In lung cancer, the most frequent HER2 mutation occurred in exon 20 (48%), with the Y772dupYVMA mutation comprising 34% of all HER2 mutations, and the least common mutation occurred in exon 21(<1%) (9). Therefore, studies of HER2 exon 21 mutations in lung cancer are rare.

## Case presentation

**Figure 1:**
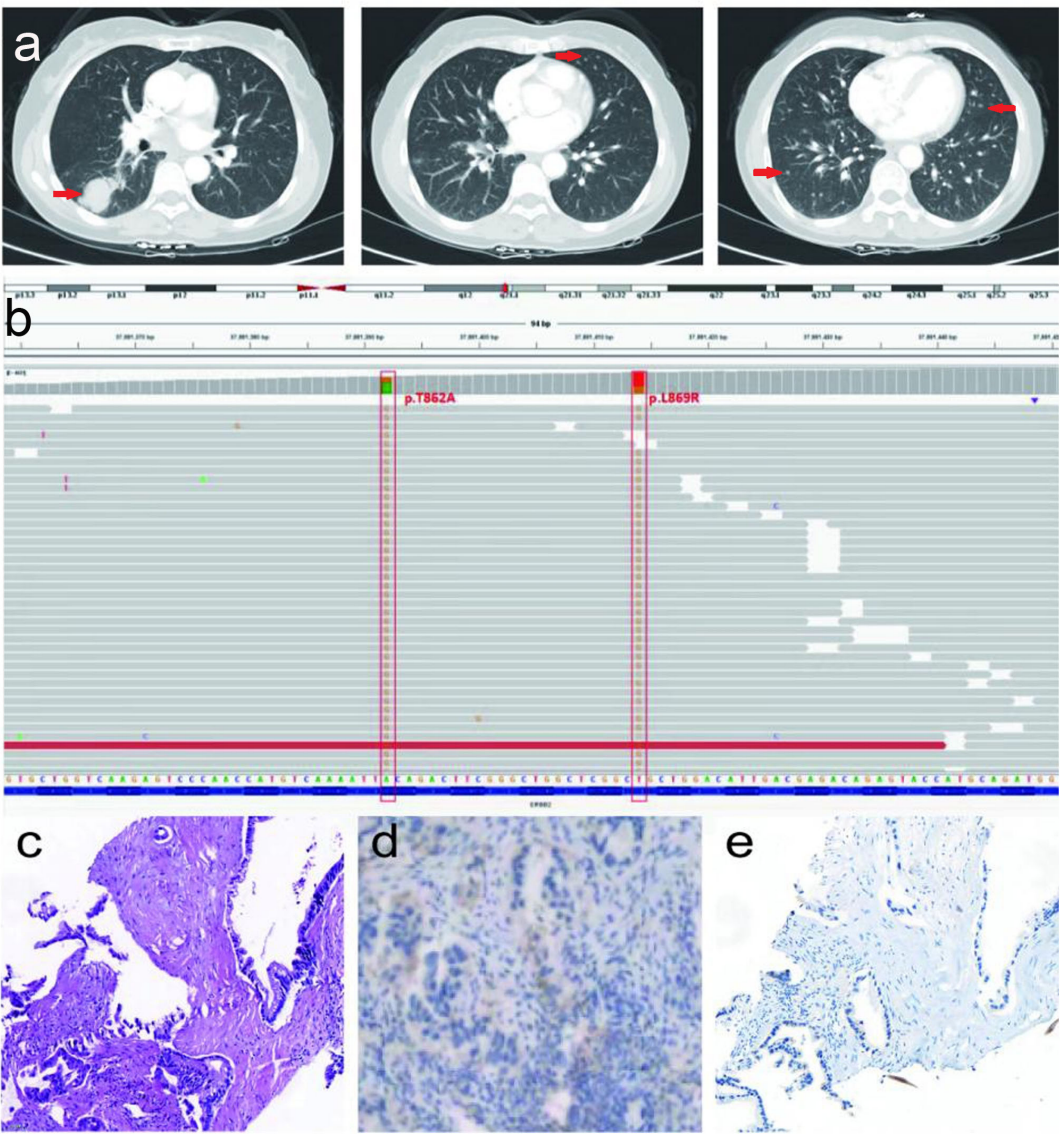
The description of a present patient with advanced-stage lung adenocarcinoma before treatment. **(A)** Serial CT scans before treatment. **(B)** NGS result of lung adenocarcinoma before treatment. **(C)** Hematoxylin and eosin staining revealed malignant cells forming glandular duct structures in the lung biopsy specimen (×200). Scale bar is 100µm. **(D)** The PD-L1 antibody type 22C3, IHC results of TPS was about 3% (×200). **(E)** Malignant cells were negative (-) for HER2(×200).

A 49-year-old female patient who had never smoked was initially diagnosed with advanced-stage lung adenocarcinoma (cT4N2M1, stage IV) on December 29, 2020. She complained of irritating dry cough symptoms followed by chest tightness. Computed tomography (CT) revealed a right lower lobe mass in the dorsal segment, multiple enlarged lymph nodes in the right hilar and mediastinum, and multiple lung metastases. Lymphatic dissemination in the right lower lobe of the lung could not be excluded (**Figure 1A**). The Eastern Cooperative Oncology Group (ECOG) performance status score was 1. Initial tumor biopsy of the primary mass showed two HER2 exon 21 insertion mutations, T8962A and L869R, with an allelic fraction of 40.00% (**Figure 1B**). Hematoxylin and eosin staining of the original aspirated tissue revealed malignant cells forming ductal structures in the lung biopsy specimen (**Figure 1C**). Based on the assessment of lung biopsy samples, programmed death-ligand 1(PD-L1) antibody type 22C3, immunohistochemical results of TPS was approximately 3% (**Figure 1D**). Simultaneously, the patient underwent immunohistochemical (IHC) detection for HER2 expression, which was negative (**Figure 1E**).

However, HER2 exon mutations are characterized by a poor response to the currently approved tyrosine kinase inhibitors (TKIs) and immune checkpoint inhibitors (ICIs). Therefore, the patient was first treated with six cycles of bevacizumab (400 mg, day 1) plus systemic chemotherapy with pemetrexed (800 mg, day 1) and carboplatin (400 mg, day 1), and her tumor burden was progressed from March 2021 to June 2021.

**Figure 2:**
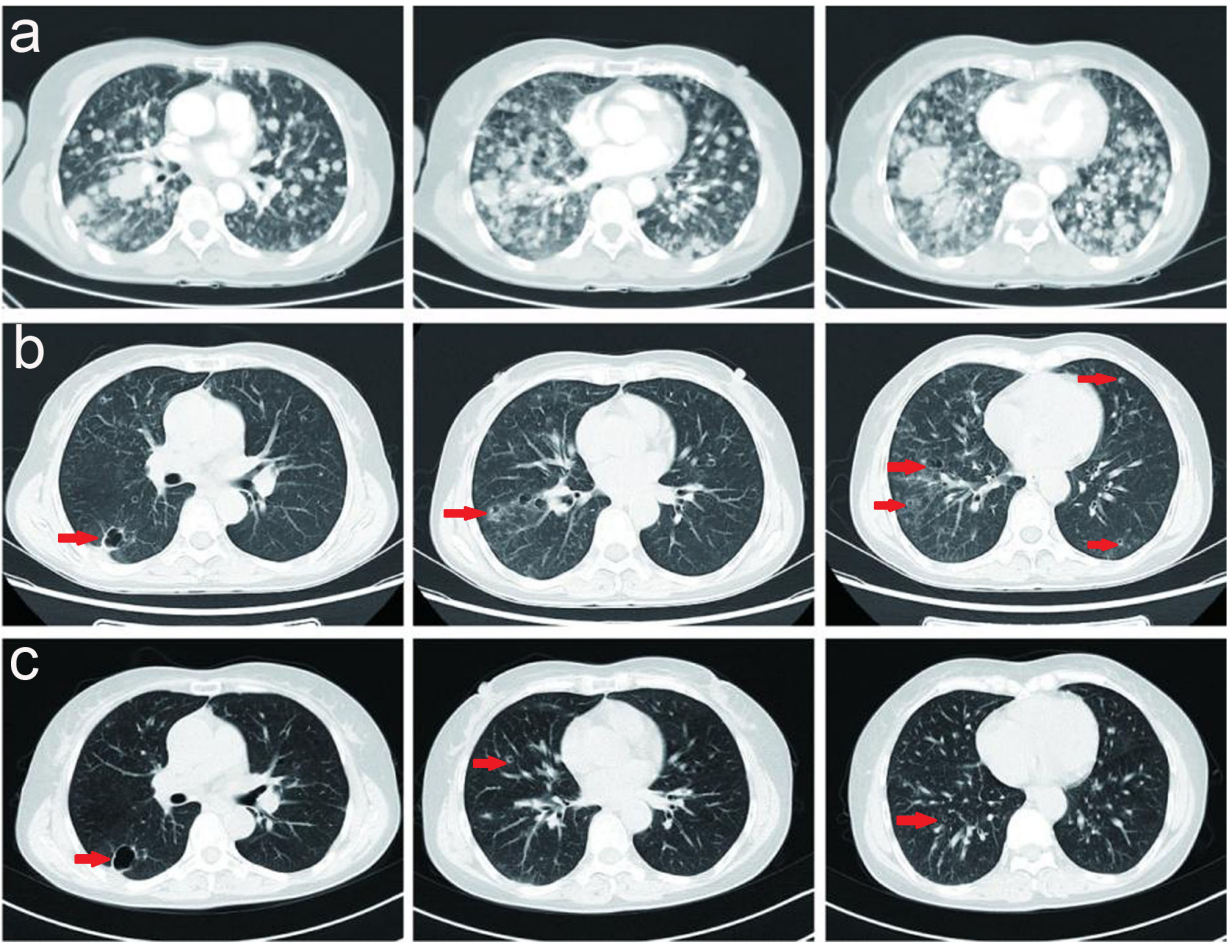
**(A)** Serial CT scans of a combination of bevacizumab (400 mg, day 1) plus pemetrexed (800 mg, day 1) and carboplatin (400 mg, day 1) for 4 cycles as a first-line treatment, and the tumor burden was progressed. **(B, C)** Serial CT scans of a combination of bevacizumab (400 mg, day 1) plus abraxane (400 mg, day 1) and nedaplatin (100 mg, day 1) for 3 cycles as a second-line treatment, and revealed prominent regression of primary lung tumors in the right lower lobe and lung-to-lung metastases.

After the failure of first-line combination treatment with bevacizumab plus carboplatin and pemetrexed in July 2021 due to progressive disease (PD) (**Figure 2A**), the patient received a combination of bevacizumab (400 mg, day 1) plus abraxane (400 mg, day 1) and nedaplatin(100 mg, day 1) for three cycles as a second-line treatment, and a CT assessment revealed prominent regression of primary lung tumors in the right lower lobe and lung-to-lung metastases from July to September 2021 (**Figures 2B, C**). She stopped chemotherapy owing to poor performance status without maintenance therapy in October 2021.

**Figure 3:**
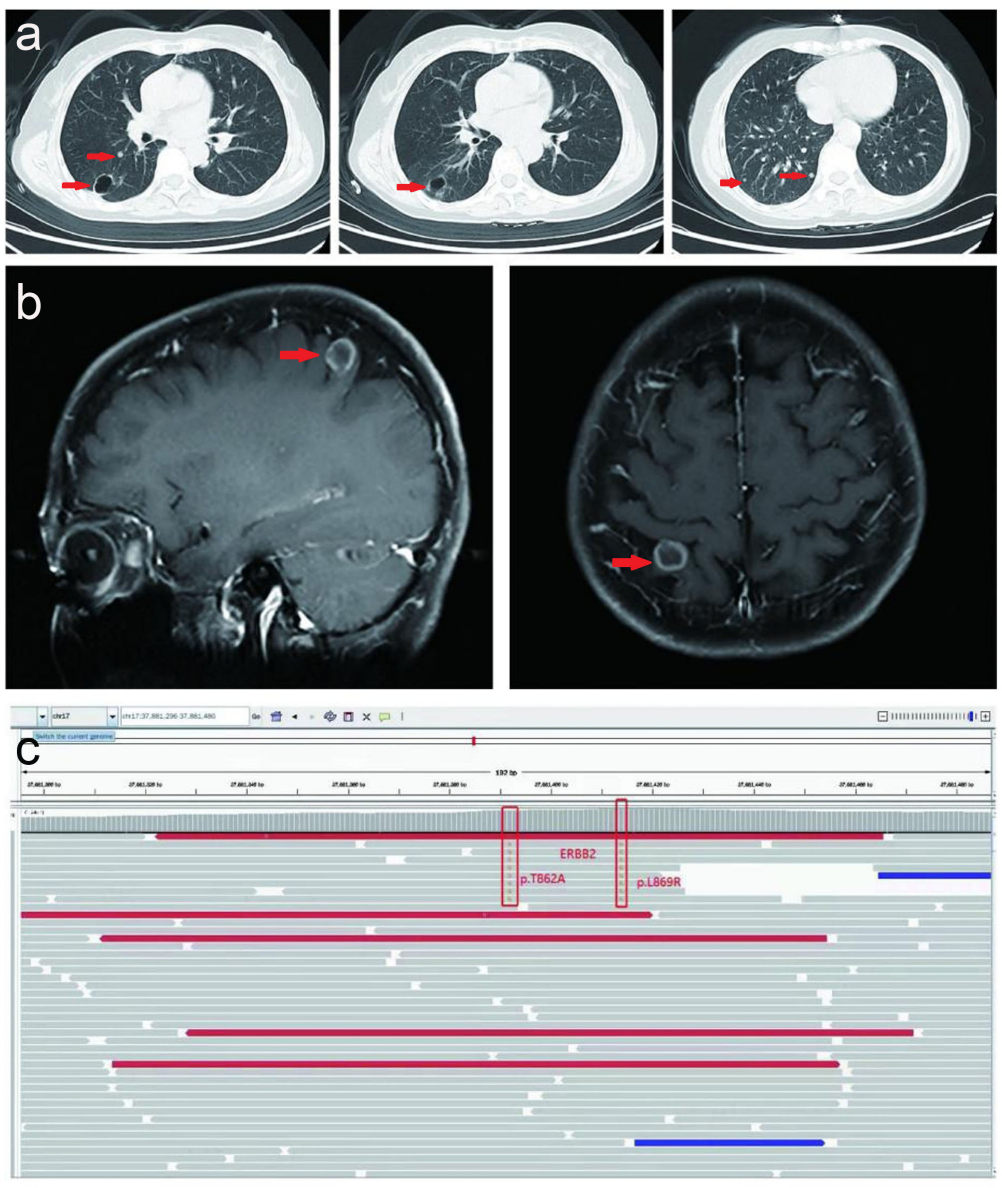
Serial CT scans and MRI. **(A)** Serial CT scans show multiple metastatic lung lesions were slightly increased after a combination of gemcitabine (1.4g day 1 and day 8) plus anlotinib (8mg, twice a day, day1-14) plus carrelizumab (200mg, day 1) for 2 cycles in June 2022. **(B)** MRI indicated and one new brain metastatic lesion was observed in June 2022. **(C)** NGS result of circulating tumor DNA before fifth-line treatment in August 2022.

Upon progression of the right lower lobar mass, multiple lung nodules and metastatic lymphadenopathies were observed after six months without subsequent treatment in April 2022. Thereafter, the patient received a combination of gemcitabine (1.4 g on day 1 and 8) plus anlotinib (8 mg, twice a day, day1-14) plus carrelizumab (200 mg, day 1) for 2 cycles, and reexamination indicated that tumor markers were elevated and the lung metastases were slightly increased compared to April 2022 (**Figure 3A**), and a new brain metastatic lesion was observed in June 2022 (**Figure 3B**).

**Figure 4:**
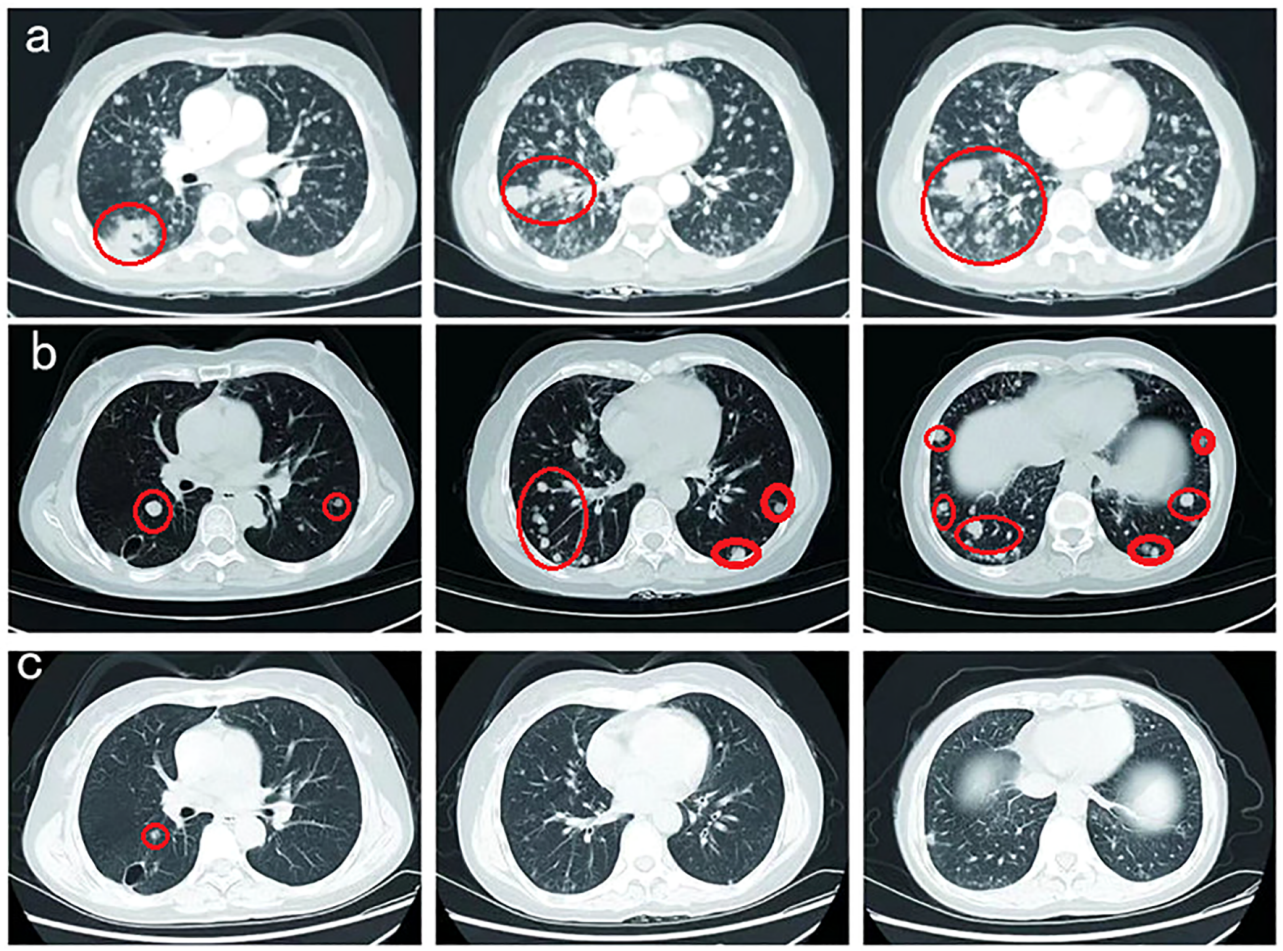
**(A)** Pulmonary nodules and metastatic lymphadenopathy were observed larger and more in August 2022. **(B)** Prominent regression of primary lung tumor in the right lower lobe and lung-to-lung metastases was observed after one month of furmonertinib treatment in September 2022. **(C)** With a double dose of furmonertinib treatment, prominent regression of lung-to-lung metastases was observed in February 2023.

Herein, the patient received radiotherapy for the metastatic brain lesion (3000cGy/300cGy/10f) and two cycles of bevacizumab (600 mg, day 1) plus abraxane (400 mg, day 1) and cisplatin (30 mg, once a day, day1-4). At the end of radiotherapy, the patient’s irritating dry cough symptoms were significantly aggravated, and pulmonary nodules and metastatic lymphadenopathy were observed in August 2022 (**Figure 4A**).

Thereafter, the patient underwent next-generation sequencing (NGS) of circulating tumor DNA to identify potential treatment opportunities. The sequencing results showed that the patient had two HER2 21 insertion mutations, T8962A and L869R, with allelic fractions of 1.28% and1.37% (**Figure 3C**). Although the National Comprehensive Cancer Network (NCCN) guidelines for NSCLC recommend antibody-coupled agents (ADCs) trastuzumab deruxtecan(T-DXd) for patients with HER2 mutant NSCLC in 2022, the patients did not choose it because of the price. After repeated discussions and considering the patient’s economic factors, the patient received furmonertinib(80 mg, once a day). Prominent regression of primary lung tumor in the right lower lobe and lung-to-lung metastases was observed after one month of treatment in September 2022 (**Figure 4B**). Her irritating dry cough was completely relieved and prominent regression of brain metastases was observed after two months of treatment. Multiple lung metastases were observed slightly larger in January 2023 in serial CT scans. Thereafter, the patient received high-dose furmonertinib (160mg, once a day), prominent regression of lung-to-lung metastases was observed after one month of treatment in February 2023 (**Figure 4C**). The patient achieved 11 months of progression-free survival (PFS) after treatment with furmonertinib (from August 2022 to July 2023), and her brain metastases remained stable. However, the patient’s disease progressed again in August 2023. Subsequently, the patient was treated with pyrrotinib (400mg, once a day) for 2 months, but the disease continued to progress. In October 2023, the patient received targeted therapy with furmonertinib again, and the multiple lung metastases in the patient were reduced and shrunk again in November 2023. However, the patient died due to hypoproteinemia combined with severe pneumonia in December 2023.

In summary, there were no treatment-related side effects that could not be tolerated during the treatment of furmonertinib. The patient’s treatment timeline and the tumor markers’ levels of the patient were shown in **Table 1**.

Table 1The patient’s treatment timeline and tumor markers’ levels of the patient.

**Table:** 

Timeline						
2021.01	2021.07	2021.10	2022.04	2022.06	2022.08	2023.08
2021.06	2021.09	2022.03	2022.05	2022.07	2023.07	2023.11
First-line treatment : 6 cycles of bevacizumab plus systemic chemotherapy with pemetrexed and carboplatin	Second-line treatment:3 cycles of bevacizumab plus abraxane and nedaplatin	The patient stopped treatment due to poor performance status.	Third-line treatment: 2 cycles of gemcitabine plus anlotinib plus carrelizumab	Fourth-line treatment:radiotherapy for the metastatic brain lesion and two cycles of bevacizumab plus Abraxane and cisplatin	Fifth-line treatment:furmonertinib(8 0mg once a day for 4 months and 160mg once a day for 7months)	Last-line treatment:2 months of pyrrotinib. Thenafter, treatment with furmonertinib for 2 months again. Eventually, the patient died of pneumonia

**Table:** 

The tumor markers’ levels					
Parameters	CA50 (0.00-25.00) IU/ml	CEA (0.00-5.10) ng/ml	CYFRA1-1 (0.10-3.30) ng/ml	NSE (0.00-16.30) ng/ml	CA125 (0.00-35.00) U/ml
2021.01.05	36.40	100.20	11.99	16.46	259.80
2021.03.01	19.40	47.94	19.52	19.96	243.90
2021.04.12	31.80	62.87	33.34	20.48	395.10
2021.05.31	58.20	81.64	46.03	34.00	771.40
2021.07.06	42.90	80.82	159.6	51.42	1024.00
2021.08.05	33.30	45.61	3.04	16.83	198.00
2021.09.13	15.50	10.51	1.70	10.68	33.48
2021.10.25	12.50	3.74	3.16	14.85	23.71
2021.12.06	11.20	4.14	2.03	13.66	26.25
2022.02.21	21.10	10.6	3.31	16.20	56.30
2022.06.27	34.20	33.00	22.00	17.80	336.00
2022.07.18	40.60	38.20	22.10	15.90	527.00
2022.10.04	31.50	30.86	2.22	9.84	111.60
2023.03.02	18.60	11.40	2.12	9.17	24.72
2023.08.19	55.50	70.82	11.97	10.19	747.9
2023.09.22	56.20	125.00	28.70	20.00	743.00
2023.10.19	32.00	41.80	19.50	20.30	476.00
2023.11.20	33.00	33.50	22.20	19.20	411.00

CA50, CEA, CYFRA1-1, NSE and CA125 were significantly reduced after the application of furmonertinib compared with the previous treatments.

## Discussion

Existing studies have shown unsatisfactory efficacy of chemotherapy and ICIs in patients with HER2-mutant NSCLC (10, 11). In our case, the patient also derived less benefit from pemetrexed-based chemotherapy than from abraxane-based chemotherapy. Carrelizumab was selected in our case and also did not benefit from ICIs. These studies highlight the urgent clinical need to develop novel therapeutic strategies for NSCLC with various HER2 alterations.

Monoclonal antibodies, including trastuzumab and pertuzumab, and tyrosine kinase inhibitors such as afatinib, dacomitinib, and neratinib, can be used as therapeutic agents targeting NSCLC harboring HER2 mutation (12), however, their efficacy is not ideal. Although anti-HER2 ADCs such as T-DXd and ado-trastuzumab emtansine(T-DM1) have also been studied to treat NSCLC patients harboring HER2 mutations with some of these drugs showing clinical benefits, their clinical applications are limited owing to price and therapeutic side effects. Therefore, it is particularly important to find effective and economical treatments for HER2 mutations in patients with NSCLC with minor side effects.

HER2 is a member of the ERBB receptor tyrosine kinase family. The ERBB receptor is activated by homodimerization or heterodimerization with other ERBB family members, especially epidermal growth factor (EGFR), which augments EGFR signaling (13). Furmonertinib is a novel third-generation EGFR TKI with central nervous system (CNS) antitumor activity that was originally developed in China. It is a promising treatment option for locally advanced or metastatic NSCLC with favorable EGFR T790M mutation status. To date, there have been no any pre-clinical studies or ongoing clinical trials regarding the efficacy and safety of furmonertinib in NSCLC patients with HER2 mutations. In this study, a 49-year-old female patient harboring two HER2 21exon mutations (T8962A and L869R) was treated with furmonertinib instead of the anti-HER2 TKIs, who achieved excellent efficacy, and prominent regression of lung metastases and brain metastases was observed after one month of treatment, which may be related to the above mechanism.

We think that furmonertinib may be a new treatment option for patients with advanced lung adenocarcinoma with HER2 exon insertion mutations, although extensive clinical studies are required to confirm this.

## Conclusion

Overall, we report the first case of a patient with NSCLC harboring two rare HER2 21 exon mutations (T8962A and L869R), who responded to furmonertinib. Based on this finding, furmonertinib may be considered as an optional treatment for NSCLC harboring HER2 21 exon mutation. Further analysis of the efficacy of HER2- targeted therapies in such cases will lay the foundation for developing optimized therapeutic regimens for patients with NSCLC with uncommon driver gene mutations.

## Data Availability

The datasets presented in this study can be found in online repositories. The names of the repository/repositories and accession number(s) can be found in the article/supplementary material.
